# Homocysteine levels in preterm infants: is there an association with intraventricular hemorrhage? a prospective cohort study

**DOI:** 10.1186/1471-2431-7-38

**Published:** 2007-11-28

**Authors:** Wendy J Sturtz, Kathleen H Leef, Amy B Mackley, Shailja Sharma, Teodoro Bottiglieri, David A Paul

**Affiliations:** 1 Department of Neonatology, Christiana Care Health System, Newark, Delaware, USA; 2 Institute of Metabolic Disease, Baylor University Medical Center, Dallas, Texas, USA; 3 Division of Neonatology, Department of Pediatrics, Thomas Jefferson University Hospital, Philadelphia, Pennsylvania, USA

## Abstract

**Background:**

The purpose of this study was to characterize total homocysteine (tHcy) levels at birth in preterm and term infants and identify associations with intraventricular hemorrhage (IVH) and other neonatal outcomes such as mortality, sepsis, necrotizing enterocolitis, bronchopulmonary dysplasia, and thrombocytopenia.

**Methods:**

123 infants < 32 weeks gestation admitted to our Level III nursery were enrolled. A group of 25 term infants were enrolled for comparison. Two blood spots collected on filter paper with admission blood drawing were analyzed by a high performance liquid chromatography (HPLC) method. Statistical analysis included ANOVA, Spearman's Rank Order Correlation and Mann-Whitney U test.

**Results:**

The median tHcy was 2.75 μmol/L with an interquartile range of 1.34 – 4.96 μmol/L. There was no difference between preterm and term tHcy (median 2.76, IQR 1.25 – 4.8 μmol/L vs median 2.54, IQR 1.55 – 7.85 μmol/L, p = 0.07). There was no statistically significant difference in tHcy in 31 preterm infants with IVH compared to infants without IVH (median 1.96, IQR 1.09 – 4.35 μmol/L vs median 2.96, IQR 1.51 – 4.84 μmol/L, p = 0.43). There was also no statistically significant difference in tHcy in 7 infants with periventricular leukomalacia (PVL) compared to infants without PVL (median 1.55, IQR 0.25 – 3.45 μmol/L vs median 2.85, IQR 1.34 – 4.82 μmol/L, p = 0.07). Male infants had lower tHcy compared to female; prenatal steroids were associated with a higher tHcy.

**Conclusion:**

In our population of preterm infants, there is no association between IVH and tHcy. Male gender, prenatal steroids and preeclampsia were associated with differences in tHcy levels.

## Background

Intraventricular hemorrhage (IVH) and periventricular leukomalacia (PVL) are significant causes of morbidity and mortality in the preterm infant population, yet aspects of the multifactorial pathophysiology leading to these CNS insults remain unclear. Petaja et al has suggested that thrombophilia may play a role in the etiology of IVH in preterm infants [1]. They identified 32% of infants with IVH as having abnormal prothrombotic factors [1]. Others have implicated activated coagulation factors in cerebral white matter damage; these activated factors are thought to cause injury by exacerbating inflammation rather than by occlusion of cerebral vessels [2]. Additionally, association has been found between hyperhomocysteinemia in term newborns and the risk of ischemic or hemorrhagic stroke [3]. The neonatal outcomes associated with abnormal coagulation factors and thrombophilia in preterm infants are not well understood. Homocysteine (Hcy), an important amino acid studied in adult thrombophilia, has not been extensively investigated as a possible contributor to preterm neonatal CNS pathologies [4].

Hyperhomocysteinemia in adults is well known to be associated with a hypercoagulable state and cardiovascular disease [4-6]. Hcy also plays an important role in pregnancy [7]. Elevated maternal total homocysteine (tHcy) levels are known to be associated with preeclampsia, prematurity, and low-birth weight [7-9]. Furthermore, maternal hyperhomocysteinemia has also been reported to be associated with placental abruption, early recurrent fetal loss, and growth retardation, as well as neural tube defects [7,10-12]. Interestingly, the tHcy level measured in pregnancy is lower than in the nonpregnant state [13,14], and maternal tHcy correlates directly with neonatal levels [15,16]. It has been suggested that tHcy levels in infants are dependent on gestational age at birth, postnatal age and neonatal diet [17-20]. Hongsprabhas characterized tHcy levels in 9 preterm versus 4 term infants and demonstrated lower levels in the preterm group [17]. Despite these documented effects of Hcy on a pregnancy and the fetus, very little is known of the role of Hcy in infants.

We theorized that an elevation of tHcy may promote thrombosis in the germinal matrix venules resulting in a post-infarction hemorrhage. We hypothesized that elevated levels of tHcy in preterm infants are associated with vascular complications, specifically IVH. The purposes of this investigation were to 1) characterize tHcy levels at birth in a preterm and term population and 2) determine if tHcy levels at birth are associated with IVH and other neonatal morbidities. This is an important investigation to undertake given that if an association exists between the neonatal tHcy levels and IVH or other neonatal morbidities, potentially modifying Hcy in pregnant mothers may affect neonatal outcome.

## Methods

This was a prospective cohort study in which infants < 32 weeks gestation who were admitted to the level III Special Care Nursery at Christiana Care Hospital from February 2002 to November 2002 were eligible. Patients were eligible regardless of family history of hematologic disorders or past pregnancy outcomes. Two blood spots were collected on filter paper specimens simultaneously with admission blood drawing. Samples were also collected for comparison on a convenience sample of term infants (≥ 37 weeks gestation) who were admitted to the Special Care Nursery during the same time period. Blood from both groups was drawn by either heel-stick procedure or arterial puncture if blood was being obtained for other purposes and was collected at < 24 hours of postnatal age. Written informed consent was obtained. The Institutional Review Board at Christiana Care Hospital approved this study.

Filter paper specimens were batched and refrigerated until transport to the Baylor Institute of Metabolic Disease where they were analyzed for tHcy by a method utilizing high performance liquid chromatography (HPLC) coupled to electrochemical (Coulometric) detection. This method of homocysteine analysis from newborn screening cards has been previously used and validated by others and ourselves [21,22].

Clinical data, including all recorded diagnoses and platelet counts, were collected from the patient's chart. In addition to IVH, other common neonatal morbidities, such as cystic PVL and necrotizing enterocolitis, which are thought to involve inflammation or vascular compromise were chosen as measured secondary outcomes. Clinical chorioamnionitis was a clinical diagnosis made by the obstetrician using maternal symptoms of fever, uterine tenderness, or foul-smelling amniotic fluid. Neonatal sepsis was defined as culture positive; necrotizing enterocolitis was defined using Bell's staging criteria, stage II or greater [23]. Admission illness severity was documented using SNAP (Score for Neonatal Acute Physiology) [24]. Bronchopulmonary dysplasia was defined as oxygen dependency at 36 weeks corrected gestational age. Thrombocytopenia was defined as a platelet count of less than 150,000. Infants were followed for all primary and secondary outcomes during their initial hospitalization until time of discharge.

All cranial ultrasounds were interpreted by a pediatric radiologist who was blinded to the infant's tHcy result. Cranial ultrasounds were obtained through the anterior fontanel using a 7.5 MHz transducer. The highest grade IVH as defined using Papile's classification was recorded [25]. Severe IVH was considered grade III and IV. Cystic PVL was defined as echolucent cysts in the periventricular white matter. Screening head ultrasounds (HUS) in our institution are typically performed in infants < 32 weeks gestation on the fourth postnatal day and at one month of age with additional ultrasounds done at the discretion of the neonatology team.

A sample size calculation determined 125 total preterm infants were necessary to enroll to show a 33% difference in tHcy in infants with IVH with a β of 0.8 and α of 0.05. We used the previously reported mean tHcy level of 3.8 ± 2.4 μmol/L reported by Hongsprabhas et al in preterm infants for calculations [17]. The historical incidence of IVH in preterm very low birth weight infants at our institution is approximately 25%. We enrolled 125 preterm infants but excluded two based on errors with the tHcy assay. Mann-Whitney U test and Spearman Rank Order Correlation were used to analyze data that was not normally distributed. ANOVA was utilized for normally distributed data. Levine's test for homogeneity was used to differentiate normally from non-normally distributed data. A *p *value of < 0.05 was considered significant. Results are reported as median and interquartile range.

## Results

Patient demographics are represented in Table 1. The median tHcy was 2.75 μmol/L with an interquartile range of 1.34 – 4.96 μmol/L. 123 preterm infants were compared to a sample of 25 term infants. There was no difference between preterm and term tHcy (median 2.76, IQR 1.25 – 4.8 μmol/L vs median 2.54, IQR 1.55 – 7.85 μmol/L, p = 0.07). In the preterm infants, there was no association between tHcy and gestational age (r = 0.15, p = 0.1) or birth weight (r = 0.14, p = 0.2) using Spearman's Rank Order Correlation.

**Table 1:** Patient demographics for all infants

	**Term** **n = 25**	**Preterm** **n = 123**
Gestational age (weeks)	37.9 ± 1.8	28.4 ± 2.4
Birth weight (g)	3343 ± 800	1188 ± 358
Male gender	20 (80%)	65 (57%)
Preeclampsia	1 (4%)	33 (27%)
Chorioamnionitis	3 (12%)	7 (6%)
Prenatal steroids	2 (8%)	74 (60%)

There was no statistically significant difference in tHcy in 31 preterm infants with IVH compared to those infants without IVH (median 1.96, IQR 1.09 – 4.35 μmol/L vs median 2.96, IQR 1.51 – 4.84 μmol/L, p = 0.43). Additionally, there was no significant difference in tHcy in the 16 infants with severe IVH compared to those infants without severe IVH (median 1.49, IQR 1.11 – 3.98 μmol/L vs median 2.83, IQR 1.42 – 4.8 μmol/L, p = 0.26). Cystic PVL was diagnosed in 7 patients and tHcy levels were not statistically significantly different in this group compared to infants without cystic PVL (median 1.55, IQR 0.25 – 3.45 μmol/L vs median 2.85, IQR 1.34 – 4.82 μmol/L, p = 0.07).

Male infants had lower tHcy compared to female (median 1.83, IQR 1.08 – 4.16 μmol/L vs median 3.26, IQR 1.95 – 5.54 μmol/L, p = 0.01). Prenatal steroids were associated with a higher tHcy (median 3.15, IQR 1.51 – 5.23 μmol/L vs median 1.82, IQR 1.14 – 3.29 μmol/L, p = 0.03) compared to infants born to mothers who did not receive steroids. Maternal preeclampsia also was associated with higher neonatal tHcy levels (median 4.32, IQR 2.31 – 6.28 μmol/L vs median 2.16, IQR 1.14 – 3.76 μmol/L, p = 0.01) compared to infants without maternal preeclampsia.

When tHcy was examined for association with other major morbidities of prematurity, there were no differences found in Hcy levels and mortality, sepsis, necrotizing enterocolitis, or bronchopulmonary dysplasia (data represented in Table 2). Table 2 also describes the tHcy levels in infants with thrombocytopenia during the first 3 days of life compared to infants without thrombocytopenia. There was no association with thrombocytopenia and tHcy level. There was also no correlation with platelet count at birth or during the 1^st ^3 days of life with tHcy (data not shown).

**Table 2:** Neonatal outcomes in 123 preterm infants versus tHcy

**Neonatal outcome**	**Number of events**	**tHcy (μmol/L) median, (IQR)**	**tHcy (μmol/L) median, (IQR)**	***p***
		**yes**	**no**	

Mortality	13	3.08 (1.83 – 4.98)	2.75 (1.25 – 4.77)	0.6
Sepsis	36	2.52 (1.09 – 3.40)	2.76 (1.42 – 4.85)	0.11
Necrotizing enterocolitis	8	2.20 (0.88 – 3.25)	2.83 (1.34 – 4.85)	0.25
Bronchopulmonary dysplasia	22	2.92 (1.14 – 5.23)	2.75 (1.42 – 4.8)	0.81
Thrombocytopenia, day of life 1	24	2.47 (1.38 – 4.37)	2.85 (1.34 – 4.85)	0.72
Thrombocytopenia, day of life 1–3	35	2.42 (1.12 – 4.29)	2.75 (1.17 – 5.19)	0.64

## Discussion

In our population of preterm infants, there was no association between tHcy measured at birth and IVH. We cannot exclude an association between tHcy level beyond birth and brain injury given that we measured values within the first 24 hours of life. Although an increased risk of other prothrombotic factors such as Factor V and prothrombin gene mutations in preterm infants with IVH was reported previously [1], IVH does not appear to be associated with an elevated tHcy in our study population as we had hypothesized.

We were able to successfully measure tHcy levels in 148 mostly preterm infants from filter paper specimens and describe the normal distribution of tHcy in our study population. The tHcy level in our infants is similar to the mean of 3.8 μmol/L described by Hongsprabhas et al in 9 Canadian preterm infants [17]. Comparable values were also reported in a US study which described a mean tHcy level of 3.49 μmol/L from term umbilical arterial samples [26]. Others have documented varying tHcy levels at birth from 3.8 to 7.9 μmol/L [13,16,27,28]; some have suggested that these differences may be due to cultural variations or differences in fortification of foods [29]. Higher tHcy values have been described in the umbilical vein as compared to umbilical artery and this may account for some of the variation as well [26].

Previous authors have suggested differences in Hcy based on gestational age with tHcy levels increasing with GA [17]. We did not identify any correlation of tHcy with gestational age in our population of infants. There also were no differences between preterm and term tHcy level. Our findings of increased tHcy in infants exposed to maternal steroids and preeclampsia may have elevated the tHcy in the preterm population contributing to our lack of difference between term and preterm tHcy. To our knowledge, our investigation examined the widest variation in GA (22–36 weeks) and the lowest mean gestation (29 ± 4 weeks) in relation to tHcy. Our data are consistent with Minet who found no correlation between tHcy and gestational age [19]. It has been previously described that elevated tHcy levels are associated with increased rates of prematurity while the natural history of maternal tHcy is to lower with pregnancy [7,14]. In 434 Chinese women, the risk of preterm birth was nearly 4-fold higher among women with preconception tHcy concentrations ≥ 12.4 μmol/L compared with women who had lower concentrations [9]. These opposing factors may also explain our lack of clear relationship between tHcy and GA.

We evaluated tHcy within the first day of life to negate any effects the neonatal diet may have. Based on our data we cannot comment on any association between tHcy levels in infants beyond the immediate postnatal period and IVH. The neonatal diet has been shown to clearly affect Hcy possibly through differences in intake of the essential vitamins necessary for folate metabolism [13,18,19]. Differences in maternal and neonatal Vitamin B12 levels account for much of the variation in tHcy. Preterm infants should not have received enteral nutrition or parenteral nutrient intake with folate or Vitamin B12 at the time of the blood draw. Therefore, the initial parenteral fluid should not have affected the tHcy value. Neonatal levels measured on the first day of life should predominantly be reflective of maternal levels and not influenced by diet or postnatal age changes.

We found lower tHcy in males as compared to females in our study sample. This has been described previously in newborn children by Refsum et al [30]. The opposite has been reported in adult males with some finding slightly higher tHcy values [4]. This finding in adults may be related to gender related differences in diet or hormonal influences which would be different in the postpubertal population compared to neonates. However, others have also reported no differences in tHcy related to gender; Minet found no difference in 123 term healthy infants as did Monsen et al in infants up to one year of age [19,20].

Additionally, prenatal steroids were associated with higher tHcy values in our neonatal population. This is the first study to describe this effect. Analogous results of elevated tHcy levels have been documented in body builders using anabolic steroids [31]. In our neonatal population, this difference in tHcy associated with prenatal steroids did not appear to be related to differences in neonatal morbidities and therefore may not be clinically important in at least the short term management of infants.

There were several limitations to our study. We only measured tHcy within 24 hours after birth. Future investigations to exclude an association between tHcy and IVH or cystic PVL might characterize tHcy beyond 24 hours but during the first several days of life, which is the highest risk period for IVH. The majority of IVH in preterm infants is known to occur in the first three days of life. Due to the small number of cases of cystic PVL in our study population, our population may have been under powered to show small differences in tHcy with cystic PVL. We also did not measure other likely important variables such as folate, vitamin B12, or maternal tHcy. However, given our limitations, our findings are similar to those recently presented by Kenet et al [32]. Their group demonstrated no association in 166 preterm infants evaluated for multiple prothrombotic factors with neonatal complications. Importantly, most tHcy samples in their study group were measured in the first one to four weeks of life, but still demonstrated no association with IVH or PVL. Our current study differs largely in the timing of the measured tHcy; we obtained all tHcy samples within 24 hours, before the majority of IVH or cystic PVL occurs.

## Conclusion

Homocysteine, while an important thrombophilic factor in certain maternal and fetal morbidities, is not clearly associated with common neonatal morbidities, specifically IVH in our population of premature infants. It remains unclear if tHcy contributes to vascular compromise that may be important in other neonatal pathologies. Further investigations are essential to fully understand the role of tHcy in neonatal coagulation and its clinical consequences.

## Abbreviations

CNS – central nervous system

GA – gestational age

Hcy – homocysteine

HPLC – high performance liquid chromatography

HUS – head ultrasound

IQR – interquartile range

IVH – intraventricular hemorrhage

PVL – periventricular leukomalacia

SNAP – Score for Neonatal Acute Physiology

tHcy – total homocysteine concentration

## Competing interests

The author(s) declare that they have no competing interests.

## Authors' contributions

DP and WS conceived of the study and participated in its design, coordination, and manuscript preparation. TB and SS participated in its design and carried out the homocysteine assays. KL and AM participated in patient enrollment and manuscript preparation. All authors read and approved the final manuscript.

## Pre-publication history

The pre-publication history for this paper can be accessed here:


